# Novel carbazole–pyridine copolymers by an economical method: synthesis, spectroscopic and thermochemical studies

**DOI:** 10.3762/bjoc.7.75

**Published:** 2011-05-19

**Authors:** Aamer Saeed, Madiha Irfan, Shahid Ameen Samra

**Affiliations:** 1 Department of Chemistry, Quaid-i-Azam University, Islamabad, Pakistan; 2 Kohat University of Science and Technology (KUST), Kohat, 26000, NWFP, Pakistan

**Keywords:** carbazole–pyridine copolymers, fluorescent materials, organic light emitting diode (OLED), photoluminescence spectra, UV spectra

## Abstract

The synthesis, as well as spectroscopic and thermochemical studies of a novel class of carbazole-4-phenylpyridine co-polymers are described. The synthesis was carried out by a simple and cheaper method compared to the lengthy methods usually adopted for the preparation of carbazole–pyridine copolymers which involve costly catalysts. Thus, two series of polymers were synthesized by a modified Chichibabin reaction, i.e., by the condensation of diacetylated *N*-alkylcarbazoles with 3-substituted benzaldehydes in the presence of ammonium acetate in refluxing acetic acid. All the polymers were characterized by FTIR, ^1^H NMR, ^13^C NMR, UV–vis spectroscopy, fluorimetry, TGA and DSC. The weight average molecular masses (*M*_w_) of the polymers were estimated by the laser light scattering (LLS) technique.

## Introduction

An immense research effort has been focused on organic semiconductors, including polymers, oligomers, dendrimers, small molecules and heavy-metal complexes, after the discovery by Burroughes et al. in 1990 [1–6] that they can be used for fabricating organic light emitting diodes (OLEDs). A number of criteria exist for employing organic materials as LEDs [7] and carbazole containing polymers are the most studied group amongst the different polymers intended for OLED applications [8–15]. Carbazole containing polymers exhibit excellent electron-donating properties together with intense luminescence, high thermal stability and excellent photoconductivity. Furthermore, the functionalization of the carbazole nucleus can easily be carried out at the 3-, 6- and 9-positions to afford a great diversity of structures [16]. Carbazole containing polymers have been greatly explored during the last few years for their applications in organic electronic devices [8]. New conjugated, fully aromatic poly-2,7-carbazole derivatives have been synthesized that exhibit high glass transition temperatures, high molecular weights and stability, and that have excellent power efficiency with low bandgaps [17–18]. Alternating poly-2,7-carbazole polymers show good thermoelectric properties [19].

Carbazole–pyridine alternate copolymers are a fascinating class of polymers on account of the fact that they take advantage of the combination of the electron-donating properties of carbazole and electron-withdrawing properties of the pyridine ring [20]. Zheng et al. have prepared a new class of copolymers that incorporate pyridine and *N*-alkyl carbazole alternatively into the main chain by oxidative-coupling copolymerization. These polymers are thermostable, highly soluble, and processable, and the fluorescence spectra of the polymers display blue light emitting properties [21].

In view of the above literature reports, it was thought to be of considerable interest to synthesize carbazole–pyridine copolymers by a route that involved the synthesis of the pyridine units as a polymer analogous reaction. To the best of our knowledge, there is no previous report of such a synthesis; the nearest precedent is the use of a Friedländer synthesis to prepare polyquinolines by Jenekhe [22]. Herein, we report a cost-effective synthesis of carbazole–pyridine copolymers in contrast to the protracted and costly methods which involve expensive catalysts.

## Results and Discussion

**Scheme 1:**
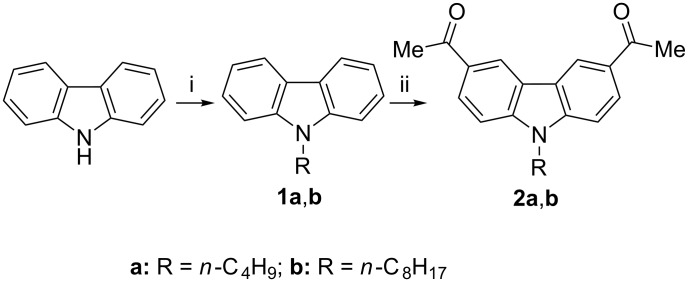
Synthesis of the monomers. i) R–Br, 50% KOH, benzene, TBAB, 80 °C, 2 h; ii) AcCl, AlCl_3_, CHCl_3_, 0 °C then rt, 3 h.

The synthetic pathway employed to prepare the monomers is outlined in Scheme 1. *N*-Butyl and *N*-octylcarbazoles were synthesized by heating a mixture of carbazole and the respective alkyl halide in a two phase system of aqueous KOH (50%) and benzene in the presence of tetrabutylammonium bromide (TBAB) as a phase-transfer catalyst [23–25]. The *N*-alkylcarbazoles were subsequently subjected to Friedel–Crafts acetylation at the active positions of carbazole, viz. 3 and 6, with anhydrous aluminum chloride in dry chloroform [26].

**Scheme 2:**
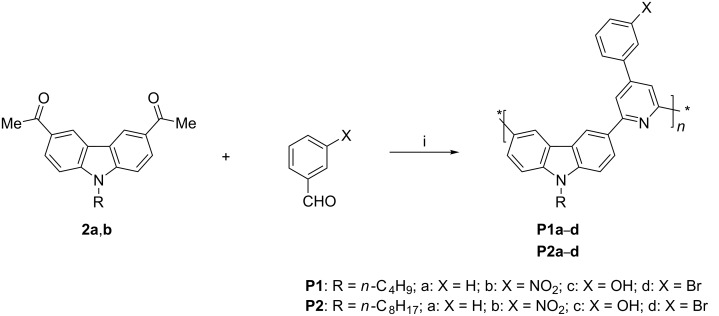
Synthesis of poly[3,6-*N*-alkylcarbazole-4-(3-substituted phenyl)pyridine-2,5-diyl]. i) CH_3_COONH_4_, CH_3_COOH, reflux, 18 h.

The monomers were purified by recrystallization. The diacetylated carbazole monomers were heated under reflux with various 3-substituted benzaldehydes and ammonium acetate in glacial acetic acid in a modified Chichibabin reaction to assemble the pyridine ring in situ and afford the desired polymers (Scheme 2) [27].

**Scheme 3:**
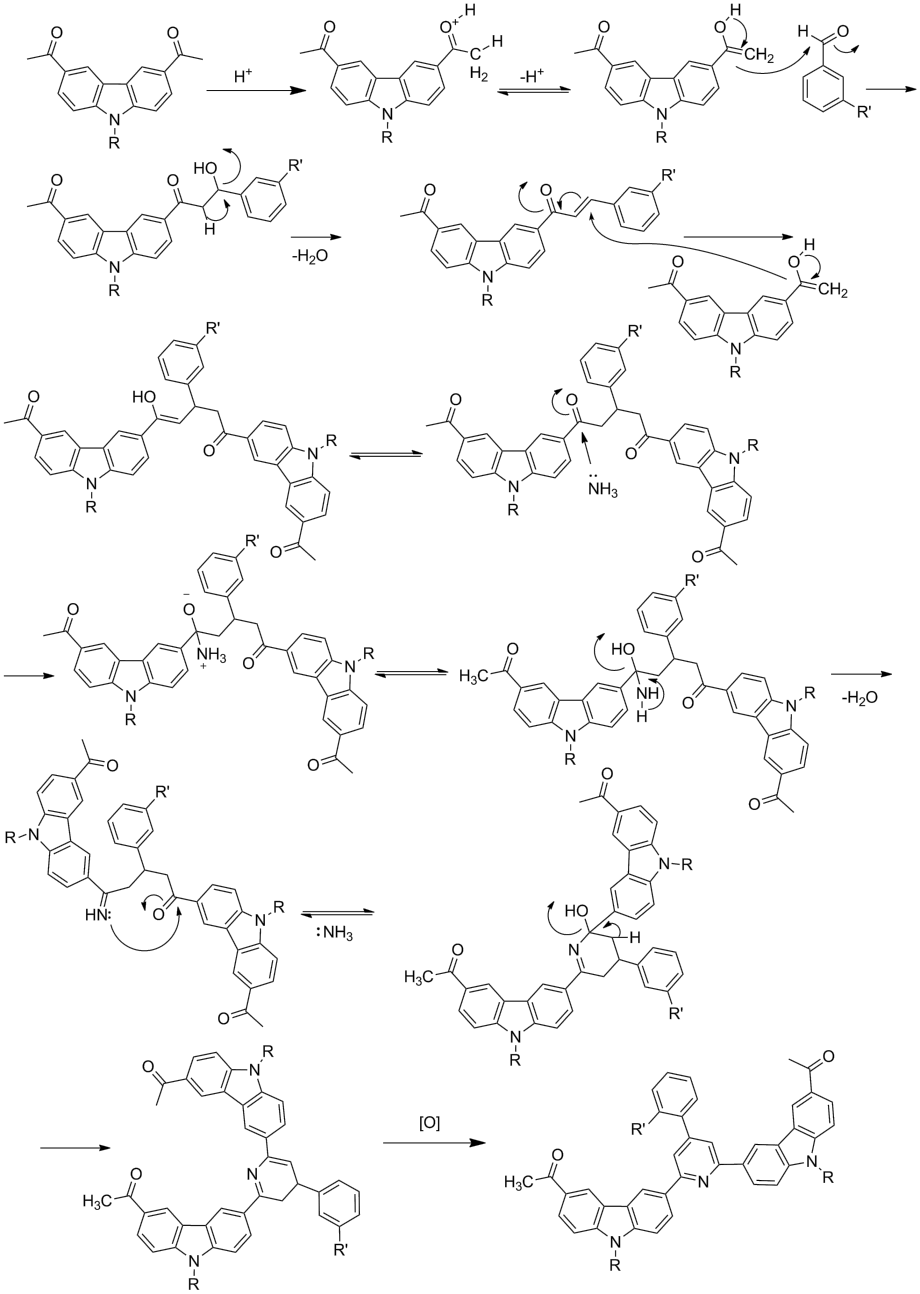
Proposed mechanism of pyridine ring formation.

The construction of the pyridine ring in situ by reaction of diacetylated carbazole monomers with different benzaldehydes and ammonium acetate may be envisaged by the complex sequence shown in Scheme 3 which involves protonation, enolization, deprotonation, dehydration, the nucleophilic addition of ammonia, intramolecular cyclization and aromatization.

The polymers were obtained by pouring the reaction mixture into distilled water, followed by filtration and washing of the residue with copious quantities of distilled water to remove completely acetic acid and salts. Finally, the polymers were dissolved in THF and re-precipitated thrice in methanol to remove oligomers. The polymers were obtained as yellow to brown powders which were then dried in a vacuum oven. The polymers were readily soluble, at room temperature, in a variety of common solvents such as tetrahydrofuran, dichloromethane, chloroform and acetone.

**Figure 1:**
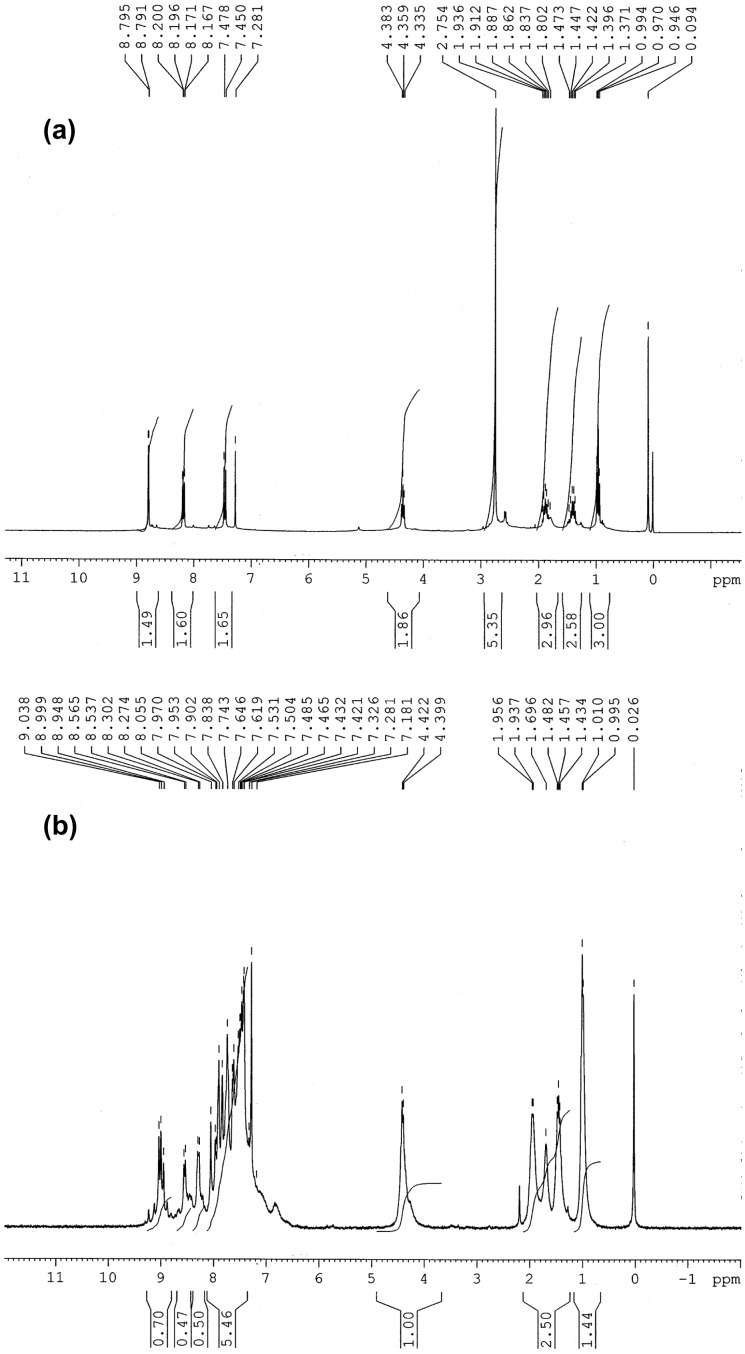
^1^H NMR spectra of (a) monomer **2a** and (b) polymer **P1a**.

All polymers were characterized on the basis of FTIR, ^1^H NMR and ^13^C NMR spectroscopic analysis. The IR spectral data of all polymers showed the characteristic peaks of C=N stretching at 1590–1584 cm^−1^, aromatic tertiary C–N stretching at 1354–1340 cm^−1^, and C–N stretching at 1153–1143 cm^−1^ showing the formation of the pyridine ring. The peaks at 3415 and 1523 cm^−1^ indicating the presence of OH- and NO_2_- groups respectively, were also observed in the corresponding polymers. Figure 1b shows the ^1^H NMR spectrum of the polymer **P1a** whilst that of corresponding monomer is shown in Figure 1a.

A comparison of the ^1^H NMR spectra of the monomer to that of the polymer indicates the disappearance of a peak due to acetyl protons at δ 2.75 ppm in addition to the appearance of a number of new peaks in the aromatic region. A complex pattern of splitting in the aromatic region and the characteristic peak broadening was also observed consistent with the in situ formation of the pyridine ring and polymerization. Polymer solutions in THF were subjected to GPC using polystyrene standards for the determination of the molecular weights, however, no elution was observed for any of the polymers. Therefore, molecular weights could not be determined by GPC, because no standard column was found to be compatible with this type of polymer. Consequently, it was decided that the molecular weights would be determined by the LLS technique, and the results are given in Table 1.

**Table 1:** Thermal data and molecular weights for polymers **P1** and **P2**.

	**P1a**	**P1b**	**P1c**	**P1d**	**P2a**	**P2b**	**P2c**	**P2d**

(*M*_w_) 10^5^ (g/mol)^a^	0.57	0.56	0.75	0.12	1.28	2.43	1.73	1.29
*T*_d_ (°C)^b^	380	300	265	300	376	295	400	295
*T*_g_ (°C)^c^	154	165	161	170	158	160	158	164

^a^Molecular weights of polymers of series **P1** and **P2** by static LLS. ^b^Decomposition temperature *T*_g_ determined by TGA at a heating rate of 10 °C/min under a N_2_ atmosphere. ^c^Glass transition temperature *T*_g_ determined by DSC at a heating rate of 10 °C/min under a N_2_ atmosphere, 2^nd^ run.

In the static laser light scattering (LLS) technique, the angular dependence of the excess absolute time-average scattered intensity, the so-called Raleigh ratio *R*_vv_(q) at a scattering angle θ is measured. The molecular weights of the polymers in a dilute solution at a concentration *C* can then be obtained on the basis of the following equation:


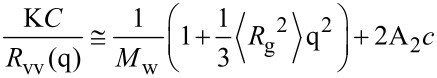


where K = 4π^2^*n*^2^(d*n*/d*C*)^2^/(*N*_A_λ_0_^4^), q = (4π*n*/λ_0_)sin(θ/2) and *N*_A_, d*n*/d*C*, *n*, λ_0_, and θ being Avogadro's number, the specific refractive index increment, the solvent refractive index, the wavelength of the light in vacuo, and the scattering angle, respectively. *M*_w_ is the weight average molar mass, A_2_ is the second virial coefficient, and <*R*_g_^2^>_z_^1/2^ is the root-mean square Z-average radius of gyration of the polymer chain.

**Figure 2:**
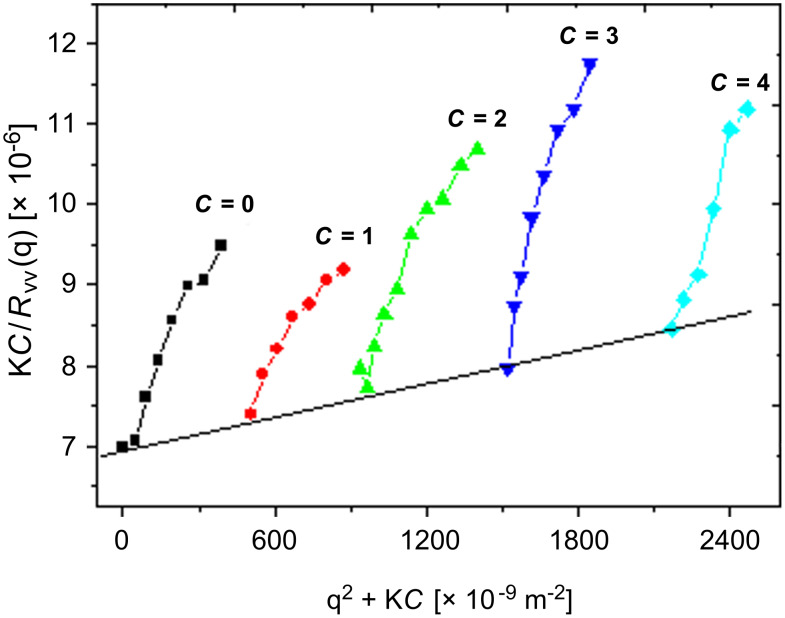
Zimm plot for **P2a** in THF at room temperature.

By measuring *R*_vv_(q) at a set of *C* and θ, M_w_, <*R*_g_>_z_ and A_2_ can be determined from a conventional Zimm plot which incorporates q and *C* extrapolation on a single grid. There are experimental points at all grid points except along the lower line (the θ = 0 line) and the left-most line (the *C* = 0) line. The values of *M*_w_ and <*R*_g_>_z_ were calculated from the slopes of [K*C*/*R*_vv_ (q)]*_C_*_→0_ vs q^2^ and [K*C*/*R*_vv_ (q)]_q→0_ vs *C* and the intercept of [K*C*/*R*_vv_ (q)]_q→0_,*_C_*_→0_, respectively. Figure 2 shows the Zimm plot of **P2a** in THF at room temperature.

### Thermochemical properties of the polymers

**Figure 3:**
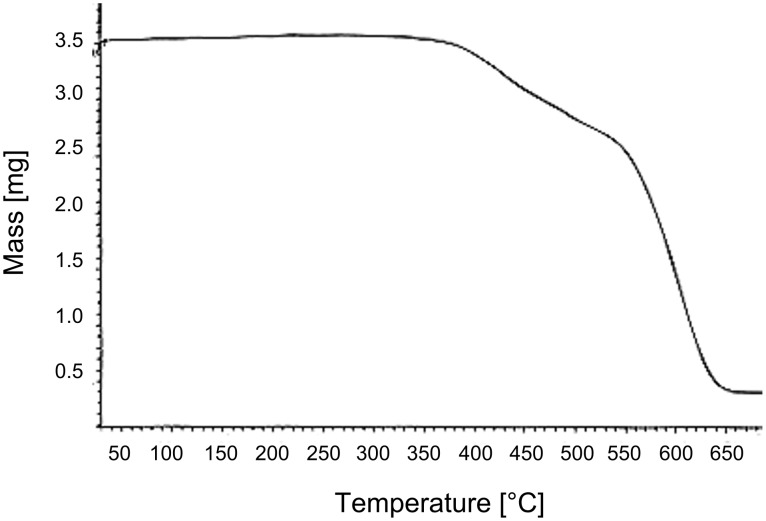
Thermogravimetric analysis curve of **P1a**.

The thermal properties of the two series of polymers, **P1** and **P2** were investigated by thermogravimetric analysis (TGA) and differential scanning calorimetry (DSC). For TGA the samples were heated (PerkinElmer Thermal Analysis System 409) from 25 °C to 85 °C at a rate of 10 °C/min and at a nitrogen gas flow rate of 80 mL/min. Polymers **P1** and **P2** lost weight gradually during the early phase of the experiment. A thermal gravimetric analysis curve of **P1a** is shown in Figure 3.

**Figure 4:**
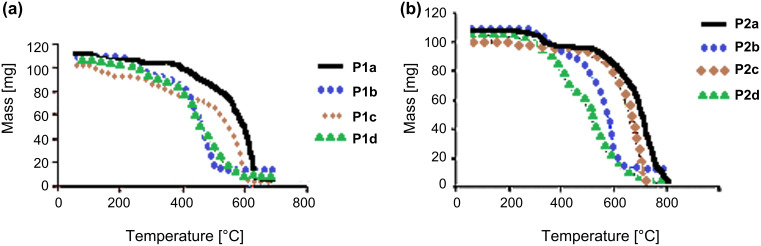
TGA curves of polymers (a) **P1** and (b) **P2**.

The thermal analysis results indicated that polymers **P1** and **P2** have a high decomposition temperature *T*_d_ and good thermal stability above 300 °C, which is very attractive for the fabrication of stable organic electroluminescent devices. TGA thermograms for polymers **P1** and **P2** are shown in Figure 4 and the results of the thermal analysis are shown in Table 1.

**Figure 5:**
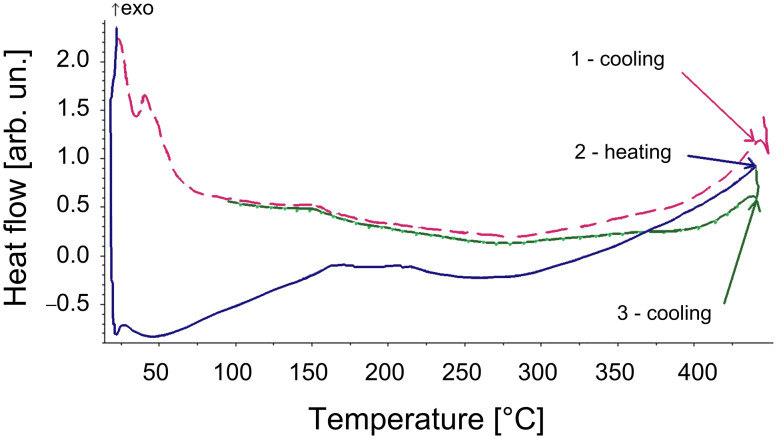
DSC graph for polymer **P1a**.

For DSC, the samples were first heated to melting, then cooled to the glassy state and reheated to determine the glass transition temperature (*T*_g_). A representative DSC graph is shown for **P1a** in Figure 5. *T*_g_ is calculated from the second heating curve. As it can be seen from the graph, *T*_g_ for the polymer is 154 °C. All the polymers show glass transition temperatures above 150 °C (Table 1).

### Photophysical properties of the polymers

The photophysical properties of the polymer, including absorption and emission spectral studies, were determined from solutions in chloroform. A summary of the measured spectroscopic data is given in Table 2.

**Table 2:** Absorption and emission spectroscopic data for polymers **P1** and **P2**.

	**P1a**	**P1b**	**P1c**	**P1d**	**P2a**	**P2b**	**P2c**	**P2d**

λ_abs,max_ [nm]	301	282	285	300	291	284	291	289
λ_em,max_ [nm]	417	424	397 (491)	421	426	435	400 (524)	431

**Figure 6:**
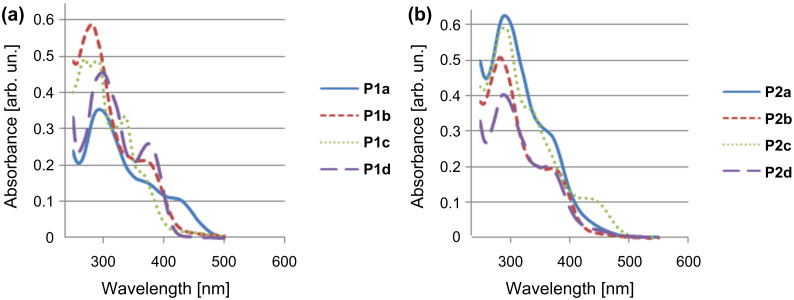
UV–vis spectra of the polymers of (a) series **P1** and (b) series **P2**.

Figure 6 shows the absorption spectra for the two series of polymers. The solution of **P1a** exhibited an absorption maximum at 301 nm along with a shoulder at a higher wavelength. Similarly, **P1b**, **P1c** and **P1d** had λ_max_ values of 282, 285 and 300 nm, respectively. Distinct shoulders are observed for all of the four polymers at higher wavelengths. Similar spectra were obtained for polymers of **P2** series. The λ_max_ values were 291, 284, 291 and 289 nm for **P2a**, **P2b**, **P2c** and **P2d**, respectively, along with distinct shoulders. The main peaks on the spectra are attributed to a π→π* transition, and the shoulders correspond to n→π* transitions in the respective materials. A comparison of the photophysical properties of the synthesized polymers may be made with structurally related polymers previously synthesized by Zheng et al. [21,28]*.* The observed differences in the absorption spectra of both polymer series arise due to the difference in alkyl chain lengths. As the chain length increases, there is a marked decrease in the bandwidth of the absorption and emission bands [29]. By comparing both series of polymers, the absorption maxima shifts to an upper limit with increasing chain length [30]. Furthermore, polymers with similar groups in both series show similar absorption maxima, but the apparent differences from one another are due to the difference in the electron-donating and electron-withdrawing nature of the substituents. The additional peak in the absorption spectrum of **P1c** is due to the optical transition attributed to the presence of the auxochromic OH group, which increases the conjugation length [31] and becomes part of extended chromophore. This effect is absent in the other compounds.

**Figure 7:**
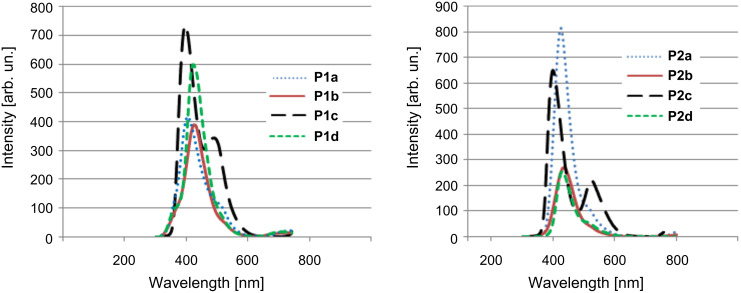
Photoluminescence spectra of polymers **P1**and **P2** in CHCl_3_ solution.

The photoluminescence spectra of the polymer solutions of series **P1** and **P2** in CHCl_3_ are shown in Figure 7. Polymers **P1a**, **P1b**, **P1c** and **P1d** show emission maxima at 417, 424, 397 and 421 nm, respectively, whereas polymers **P2a**, **P2b**, **P2c** and **P2d** show maxima at 426, 435, 400 and 431 nm, respectively. Polymers **P1c** and **P2c** having hydroxyl functionality show an extra emission band at 491 and 524 nm, respectively. These suggest that the polymer solutions in CHCl_3_ should emit blue–green light.

**Figure 8:**
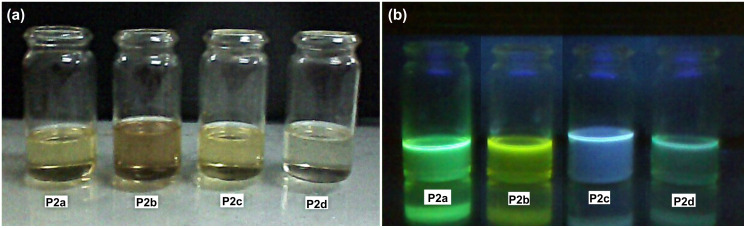
(a) Polymers of **P2** series in visible light; (b) observed fluorescence (CHCl_3_ dilute solutions) under UV irradiation (254 nm).

The emission colors of dilute solutions of the polymer samples were also observed under UV light and are shown in Figure 8.

## Conclusion

A new class of polymeric materials that contain alternating hole transporting and electron transporting moieties of carbazole and pyridine, respectively, has been synthesized by a simple and inexpensive method. The polymers are highly soluble in most common solvents, which makes them easily to process by spin coating methods. These materials can be considered as potential candidates for application as OLEDs. The synthesis of further polymers of this novel class is underway.

## Experimental

Melting points were recorded using a digital Gallenkamp (Sanyo) model MPD BM 3.5 apparatus and are uncorrected. ^1^H and ^13^C NMR spectra were determined in deuterochloroform solutions using a Bruker AM-300 spectrophotometer. FTIR spectra were recorded on an FTS 3000 MX spectrophotometer. Mass spectra (EI, 70eV) were obtained on a GC–MS instrument of Agilent technologies. UV–vis and fluorescence spectra were recorded on a Perkin Elmer Lambda 20 UV–visible spectrophotometer and a Perkin Elmer LS 55 fluorescence spectrophotometer, respectively.

Molecular weights of the polymers were determined by the laser light scattering (LLS) technique using a commercial light-scattering spectrometer (ALV/SP-150 equipped with an ALV-5000 multi-τ digital time correlator) with a solid-state laser (ADLAS DPY 425II, output power ≈ 400 mW at λ = 532 nm) as the light source. Thermogravimetric analysis (TGA) was performed with a PerkinElmer Thermal analysis System 409. Differential scanning calorimetry (DSC) measurements were performed using Bruker Reflex II thermosystem. The TGA and DSC measurements were recorded under a nitrogen atmosphere at a heating rate of 10 °C/min. Elemental analyses were performed on CHNS 932 LECO instrument.

Carbazole, 1-bromobutane, 1-bromooctane, tetrabutylammonium bromide (TBAB) and aluminum chloride were commercial products from Fluka; acetyl chloride, benzaldehydes and ammonium acetate were purchased from the Aldrich Chemical Company. Glacial acetic acid was purchased from Riedel de-Haën.

### 

#### Synthesis of 9-butylcarbazole (**1a**)

A mixture of carbazole (8.35 g, 50 mmol), 1-bromobutane (7.13 g, 52 mmol), benzene (25 mL), 50% KOH aqueous solution (60 mL), tetrabutylammonium bromide (TBAB, 0.4 g, 1.25 mmol) was stirred at 80 °C for 2 h. The reaction mixture was cooled to room temperature. The benzene layer was separated, diluted with 20 mL of ethyl acetate, washed with three 50 mL portions of water and dried over anhydrous MgSO_4_. Solvents were removed in vacuo to give a viscous oil, which solidified on standing at room temperature to afford a white solid. Recrystallization from ethanol gave **1a** as white needles (92%). Mp 54–56 °C; IR (KBr, υ/cm^−1^): 1640, 1350, 1153; ^1^H NMR (CDCl_3_, 300 MHz): δ 8.16 (d, *J* = 7.8 Hz, 2H), 7.51 (m, 4H), 7.28 (d, *J* = 7.6 Hz, 2H), 4.35 (t, *J* = 7.2 Hz, 2H), 1.90 (m, 2H), 1.45 (m, 2H), 0.99 (t, *J* = 7.2 Hz, 3H); ^13^C NMR (CDCl_3_, 75 MHz): δ 140.7, 125.5, 122.8, 120.3, 118.7, 108.6, 42.8, 31.17, 20.62, 13.95; Elemental analysis: Calcd C: 86.0%, H: 7.6%, N: 6.2%; found C: 85.8%, H: 7.5%, N: 6.1%.

#### 9-Octylcarbazole (**1b**)

The compound **1b** was synthesized by the same procedure as for **1a** using carbazole (8.35 g, 50 mmol), 1-bromooctane (10.0 g, 52 mmol), benzene (25 mL), and TBAB (0.4 g, 1.25 mmol). The product was obtained as a light-yellow oil (87%). IR (NaCl cell, υ/cm^−1^): 1630, 1340, 1147; ^1^H NMR (CDCl_3_, 300 MHz): δ 8.35 (d, *J* = 7.8 Hz, 2H), 7.69 (m, 4H), 7.59 (d, *J* = 8.1 Hz, 2H), 4.41 (t, *J* = 7.2 Hz, 2H), 2.03 (m, 2H), 1.51 (m, 5H), 1.14 (t, *J* = 7.2 Hz, 3H); ^13^C NMR (CDCl_3_, 75 MHz): δ 140.5, 125.8, 123.0, 120.5, 118.9, 108.9, 43.1, 32.1, 29.6, 29.4, 29.2, 27.5, 22.9, 14.4; Elemental analysis: Calcd C: 85.9%, H: 9.0%, N: 5.0%; found C: 85.8%, H: 8.9%, N: 5.0%.

#### 3,6-Diacetyl-9-butylcarbazole (**2a**)

Aluminum chloride, (4.0 g, 3 mmol) and acetyl chloride, (2.35 g, 3 mmol) were added successively to 10 mL of dry chloroform. The mixture was stirred for 10 min at 0 °C to obtain a clear solution. A solution of (4.46 g, 2 mmol) of **1a** in 10 mL of dry chloroform was added dropwise to the above solution at 0 °C during 15 min. The reaction mixture was stirred at room temperature for 3 h. After the completion of the reaction (TLC monitoring), the reaction mixture was poured into a stirred solution of 10% HCl (50 mL). The organic layer was separated, washed with distilled water three times and dried over anhydrous Na_2_SO_4_. The solvent was removed in vacuo to give a solid which was recrystallized from ethanol to afford **2a** (85%) as dull green crystals with a moldy bread smell. Mp 145 °C; IR (KBr, υ/cm^−1^): 2907, 1718, 1655, 1348, 1145; ^1^H NMR (CDCl_3_, 300 MHz): δ 8.79 (s, 2H), 8.18 (dd, *J* = 8.7 Hz, 2H), 7.45 (d, *J* = 8.4 Hz, 2H), 4.35 (t, *J* = 7.2 Hz, 2H), 2.75 (s, 6H), 1.87 (m, 2H), 1.42 (m, 2H), 0.97 (t, *J* = 7.2 Hz, 3H); ^13^C NMR (CDCl_3_, 75 MHz): δ 197.4, 143.9, 129.6, 127.0, 122.8, 122.0, 108.9, 43.3, 31.0, 26.6, 20.4, 13.8; GC–MS (*m*/*z*): 307 (M^.+^), 264 (100%), 221, 178, 43, 29.

#### 3,6-Diacetyl-9-octylcarbazole (**2b**)

The compound **1b** was synthesized by the same procedure as for **2a** from AlCl_3_, 4.00 g (3 mmol), acetyl chloride, 2.35 g (3 mmol) to afford **1b** 5.58 g (2 mmol) in 88% yield as dull green crystals. Mp 139 °C; IR (KBr, υ/cm^−1^): 2939, 1715, 1640, 1350, 1140; ^1^H NMR (CDCl_3_, 300 MHz): δ 8.75 (s, 2H, Ar-1,1’), 8.18 (dd, 2H, *J* = 8.4 Hz, Ar-2,2’), 7.45 (d, 2H, *J* = 8.7 Hz, Ar-3,3’), 4.34 (t, 2H, *J* = 7.2 Hz, H-a), 2.75 (s, 6H, COCH_3_), 1.86 (m, 2H, H-b), 1.30 (m, 10H, H-c,d,e,f,g), 0.86 (t, 3H, *J* = 6.9 Hz, H-h); ^13^C NMR (CDCl_3_, 75 MHz): δ 198.1, 142.4, 128.9, 127.0, 121.8, 120.3, 109.9, 43.1, 31.0, 29.3, 28.9, 27.2, 22.4, 13.8; GC–MS (*m*/*z*): 363 (M^.+^), 264 (100%), 348, 221, 178, 43, 29.

#### General procedure for the synthesis of polymers **P1** and **P2** (**a**–**d**)

Ammonium acetate (10 mmol) was added portionwise to a stirred equimolar mixture of 3,6-diacetyl-*N*-alkylcarbazole and benzaldehyde in acetic acid (10 mL). The reaction mixture was heated under reflux for 18 h. Upon completion of reaction (TLC monitoring), the reaction mixture was cooled to room temperature, poured into distilled water (20 mL) and stirred for 5 min. The precipitated solid was filtered and washed well with copious quantities of distilled water to remove acetic acid and salts completely. The resulting solid was dissolved in THF and precipitated by adding methanol to remove the oligomers. The precipitation process was repeated three times and the polymers obtained were dried in a vacuum oven at 70 °C overnight.

#### Poly{[*N*-butylcarbazole-3,6-diyl][4-phenylpyridine-2,6-diyl]} (**P1a**)

Yellow solid; yield: 44%; IR (KBr, υ/cm^−1^): 3055, 2930, 2860, 1658, 1584, 1326, 1153; ^1^H NMR (CDCl_3_, 300 MHz): δ 8.94 (s, Ar-1), 8.55 (dd, *J* = 8.4 Hz, Ar-2), 8.28 (d, *J* = 8.4 Hz, Ar-H), 8.0–7.5 (m, Ar H), 4.4 (t, 2H, *J* = 6.9 Hz, H-a), 1.7 (m, 4H, H-b,c), 1.0 (t, 3H, *J* = 6.9 Hz, H-d); ^13^C NMR (CDCl_3_, 75 MHz): δ 148.6, 146.2, 144.1, 142.3, 139.8, 137.2, 129.0, 128.7, 127.5, 120.5, 119.1, 116.7, 109.5, 33.2, 29.9, 29.2, 28.7, 27.2, 21.9, 14.8; Elemental analysis: Calcd C: 86.6%, H: 5.8%, N: 7.4%; found C: 84.3%, H: 5.9%, N: 7.1%.

#### Poly{[*N*-butylcarbazole-3,6-diyl][4-(3-nitrophenyl)pyridine-2,6-diyl]} (**P1b**)

Yellow powder; yield: 49%; IR (KBr, υ/cm^−1^): 3035, 2930, 2890, 1656, 1585, 1523, 1345, 1145, 800; ^1^H NMR (CDCl_3_, 300 MHz): δ 8.96 (s, Ar-8), 8.58 (s, Ar-7), 8.27 (m, Ar-H), 8.0 (d, *J* = 7.8 Hz, Ar-5), 7.92 (s, Ar-4), 7.66 (d, *J* = 8.1 Hz, Ar-3), 7.54 (m, Ar-H), 4.4 (t, 2H, *J* = 7.1 Hz, H-d), 1.9 (m, 2H, H-c), 1.46 (m, 2H, H-b), 1.0 (t, 3H, *J* = 7.2 Hz, H-a); ^13^C NMR (CDCl_3_, 75 MHz): δ 148.9, 144.5, 142.0, 137.1, 134.3, 130.5, 130.0, 127.8, 124.9, 124.5, 123.3, 122.1, 108.7, 33.5, 29.3, 27.6, 22.6, 14.8; Elemental analysis: Calcd C: 77.3%, H: 5.0%, N: 10.0%; found C: 76.9%, H: 4.7%, N: 9.8%.

#### Poly{[*N*-butylcarbazole-3,6-diyl][4-(3-hydroxyphenyl)pyridine-2,6-diyl]} (**P1c**)

Dark brown powder; yield: 40%; IR (KBr, υ/cm^−1^): 3415, 3110, 2955, 2860, 1663, 1584, 1354, 1146; ^1^H NMR (CDCl_3_, 300 MHz): δ 8.79 (s, Ar-1), 8.21 (dd, *J* = 1.9, 9.0 Hz, Ar-2), 7.47–7.41 (m, Ar-H), 7.31–7.18 (m, Ar-H), 7.02–6.99 (m, Ar-H), 5.10 (s, OH), 4.34 (t, 2H, *J* = 9.0 Hz, H-a), 1.93–1.79 (m, 2H, H-b), 1.44–1.35 (m, 2H, H-c), 0.97 (t, 3H, *J* = 9.0 Hz, H-d); ^13^C NMR (CDCl_3_, 75 MHz): δ 150.1, 147.1, 144.3, 141.6, 139.7, 137.8, 134.4, 131.1, 129.3, 126.2, 125.4, 122.9, 120.4, 117.1, 109.3, 36.2, 29.3, 28.5, 28.1, 25.5, 22.2, 14.6; Elemental analysis: Calcd C: 83.0%, H: 5.6%, N: 7.1%; found C: 82.6%, H: 5.3%, N: 6.8%.

#### Poly{[*N*-butylcarbazole-3,6-diyl][4-(3-bromophenyl)pyridine-2,6-diyl]} (**P1d**)

Yellow precipitate; yield: 66%; *R*_f_: 0.45; IR (KBr, υ/cm^−1^): 3065, 2960, 2890, 1652, 1596, 1340, 1143, 664; ^1^H NMR (CDCl_3_, 300 MHz): δ 8.91 (s, Ar-1), 8.28 (dd, *J* = 8.7 Hz, Ar-2), 7.78 (d, *J* = 4.2 Hz, Ar-H), 7.55 (m, Ar-H), 7.33 (at, Ar-H), 4.38 (t, 2H, *J* = 7.2 Hz, H-a), 1.91 (m, 2H, H-b), 1.42 (m, 2H, H-c), 0.98 (t, 3H, *J* = 7.2 Hz, H-d); ^13^C NMR (CDCl_3_, 75 MHz): δ 145.6, 143.2, 136.5, 134.1, 133.7, 131.4, 130.5, 130.3, 127.5, 126.2, 123.2, 123.0, 122.6, 109.1, 32.7, 29.1, 27.3, 22.4, 14.8; Elemental analysis: Calcd C: 71.5%, H: 4.6%, N: 6.1%; found C: 69.3%, H: 4.3%, N: 6.0%.

#### Poly{[*N*-octylcarbazole-3,6-diyl][4-phenylpyridine-2,6-diyl]} (**P2a**)

Yellow powder; yield: 39%; IR (KBr, υ/cm^−1^): 3060, 2950, 2862, 1651, 1590, 1326, 1145; ^1^H NMR (CDCl_3_, 300 MHz): δ 8.94 (s, Ar-1), 8.54–7.47 (m, Ar Hs), 4.38 (t, 2H, H-a), 1.93 (m, 2H, H-b), 1.27 (m, 10H, H-c,d,e,f,g), 0.88 (t, 3H, H-h); ^13^C NMR (CDCl_3_, 75 MHz): δ 150.6, 148.3, 147.1, 144.4, 139.8, 136.5, 129.0, 127.3, 126.2, 120.7, 118.1, 116.5, 110.5, 35.2, 30.1, 29.2, 28.7, 27.2, 21.9, 14.8; Elemental analysis: Calcd C: 86.5%, H: 6.9%, N: 6.5%; found C: 83.3%, H: 6.7%, N: 6.1%.

#### Poly{[*N*-octylcarbazole-3,6-diyl][4-(3-nitrophenyl)pyridine-2,6-diyl]} (**P2b**)

Yellow powder; yield: 46%; IR (KBr, υ/cm^−1^): 3070, 2950, 2840, 1654, 1586, 1345, 1145, 802; ^1^H NMR (CDCl_3_, 300 MHz): δ 8.96 (s, Ar-8), 8.58 (s, Ar-7), 8.33–8.22 (m, Ar-H), 8.0 (d, *J* = 7.8 Hz, Ar-H), 7.92 (s, Ar-H), 7.66 (at, Ar-H), 7.5 (d, *J* = 8.7 Hz, Ar-H), 4.4 (t, 2H, *J* = 6.9 Hz, H-a), 1.93 (m, 12H, H-b,c,d,e,f,g), 0.86 (t, 3H, *J* = 6.9 Hz, H-h); ^13^C NMR (CDCl_3_, 75 MHz): δ 148.7, 144.1, 141.0, 136.9, 134.3, 130.1, 130.0, 127.6, 124.6, 124.5, 123.1, 122.4, 109.5, 31.7, 29.3, 29.1, 28.4, 27.2, 22.6, 14.0; Elemental analysis: Calcd C: 78.3%, H: 6.1%, N: 8.8%; found C: 77.1%, H: 5.9%, N: 8.2%.

#### Poly{[*N*-octylcarbazole-3,6-diyl][4-(3-hydroxyphenyl)pyridine-2,6-diyl]} (**P2c**)

Brownish-black solid; yield: 42%; IR (KBr, υ/cm^−1^): 3345, 3085, 2925, 2840, 1640, 1589, 1350, 1152; ^1^H NMR (CDCl_3_, 300 MHz): δ 8.94 (s, Ar-H), 8.89 (m, Ar-H), 8.84–8.81 (m, Ar-H), 8.22–8.17 (m, Ar-H), 7.49–7.46 (m, Ar-H), 5.31 (s, OH), 4.36 (t, 2H, *J* = 7.1 Hz, H-a), 2.20 (m, 2H, H-b), 1.45–1.34 (m, 10H, H-c,d,e,f,g), 0.87 (t, 3H, *J* = 7.2 Hz, H-h); ^13^C NMR (CDCl_3_, 75 MHz): δ 153.1, 145.2, 144.3, 141.1, 139.0, 137.2, 134.4, 132.1, 129.3, 127.2, 125.3, 122.6, 120.2, 117.3, 108.2, 39.2, 29.4, 28.6, 28.2, 26.3, 22.5, 15.1; Elemental analysis: Calcd C: 83.4%, H: 6.7%, N: 6.2%; found C: 82.1%, H: 6.4%, N: 6.0%.

#### Poly{[*N*-octylcarbazole-3,6-diyl][4-(3-bromophenyl)pyridine-2,6-diyl]} (**P2d**)

Dark yellow precipitates; yield: 40%; *R*_f_: 0.47; IR (KBr, υ/cm^−1^): 3020, 2960, 2855, 1651, 1587, 1343, 1144, 668; ^1^H NMR (CDCl_3_, 300 MHz): δ 8.92 (s, Ar-1), 8.28 (d, *J* = 7.5 Hz, Ar-H), 7.92–7.88 (m, Ar-H), 7.63–7.49 (m, Ar-H), 7.33 (at, Ar-H), 4.37 (t, 2H, *J* = 6.6 Hz, H-a), 1.92 (m, 2H, H-b), 1.36–1.26 (m, 10H, H-c,d,e,f,g), 0.87 (t, 3H, *J* = 6.9 Hz, H-h); ^13^C NMR (CDCl_3_, 75 MHz): δ 144.0, 142.2, 137.2, 133.1, 130.8, 130.4, 130.4, 130.3, 127.5, 127.3, 123.2, 123.1, 123.0, 122.2, 109.3, 31.7, 29.3, 29.1, 28.9, 27.3, 22.6, 14.1; Elemental analysis: Calcd C: 73%, H: 5.6%, N: 5.5%; found C: 70%, H: 5.4%, N: 5.2%.
